# Predictive genetic testing for Motor neuron disease: time for a guideline?

**DOI:** 10.1038/s41431-022-01093-y

**Published:** 2022-04-05

**Authors:** Alisdair McNeill, Maria-del-Mar Amador, Hilary Bekker, Angus Clarke, Ashley Crook, Cathy Cummings, Alison McEwen, Christopher McDermott, Oliver Quarrell, Alessandra Renieri, Jennifer Roggenbuck, Kristiana Salmon, Alexander Volk, Jochen Weishaupt, International Alliance of ALS/MND Associations

**Affiliations:** 1 Department of Neuroscience, the University of Sheffield, 385A Glossop Road, Sheffield, S10 2HQ UK; 2 Département de Neurologie, Centre de Référence SLA de Paris, Assistance Publique-Hôpitaux de Paris, Sorbonne Université Hospital Pitié-Salpêtrière, Paris, France; 3 Leeds Unit of Complex Intervention Development (LUCID), Leeds Institute of Health Science, University of Leeds, Leeds, UK; 4 Medical Genetics, School of Medicine, Cardiff University, Wales, CF10 3AT UK; 5 Centre for MND Research, Department of Biomedical Science, Faculty of Medicine and Health Sciences, Macquarie University, Sydney, NSW Australia; 6 International Aliance of ALS/MND Associations, Northampton, UK; 7 Graduate School of Health, University of Technology Sydney, Chippendale, NSW Australia; 8 Medical Genetics, University of Siena, Siena, Italy; 9 Department of Neurology and Internal Medicine, The Ohio State University Wexner Medical Center, Columbus, OH 43212 USA; 10 Department of Neurology & Neurosurgery, Montreal Neurological Institute-Hospital, McGill University, Montreal, QC Canada; 11 Institute of Human Genetics, University Medical Center Hamburg Eppendorf (UKE), Martinistr. 52, 20246 Hamburg, Germany; 12 Division of Neurodegeneration, Department of Neurology, Mannheim Center for Translational Neurosciences (MCTN), Medical Faculty Mannheim, Heidelberg University, Theodor-Kutzer-Ufer 1-3, 68167 Mannheim, Germany

Predictive (presymptomatic) testing refers to the situation where a person at risk of inheriting a specific condition requests a genetic test to clarify their status. This most commonly occurs in familial cancer, cardiac and neurodegenerative disorders. People seek predictive testing for a variety of reasons including to reduce uncertainty, enable financial planning or access reproductive medicine options [1].

Until recently, predictive testing for motor neuron disease (MND, also known as amyotrophic lateral sclerosis (ALS)) was available to only a small proportion of families who had a known disease causing genetic variant in a limited group of causal genes (e.g. *SOD1*) [2]. However, the application of newer genomic technologies has identified many more genes linked to MND (e.g. *c9orf72*) [3]. Even in the absence of a family history of MND, comprehensive genomic approaches (*c9orf72* expansion testing, followed by gene panel testing) can identify a causal genetic variant in around 20% of MND probands of Western European ethnicity [3]. This significantly increases the number of families for whom predictive testing is available. In our clinical experience, this has led to a noticeable increase in referrals to Neurogenetic or Clinical Genetic services for genetic counselling and testing. It is also noteworthy that contemporary clinical trials of disease modifying therapies for MND have started to recruit presymptomatic gene variant carriers, potentially creating an additional motivation to pursue predictive genetic testing [4].

There are few studies of predictive testing for MND. A cohort study from France identified an increase in requests for predictive MND genetic testing in recent years [5]. The majority of predictive test requests related to the *c9orf72* gene. The motivation to pursue testing included informing life decisions, understanding reproductive risk and being able to inform relatives of their risk of inheriting a predisposition to developing MND. Qualitative interview studies of people who have undergone predictive genetic testing for MND confirm the potential for significant psychological distress associated with the testing process [6].

Globally, Clinical Genetic and Neurogenetic services undertake predictive testing for MND using a pathway based on established practice for Huntington disease (HD) families [4]. HD is an autosomal dominant, fatal neurodegenerative disorder affecting movement and cognition. In HD, a trinucleotide repeat expansion in a single gene (*Htt*) is associated with disease [7]. The clinical manifestations and natural history of HD are relatively homogenous, with a recognised prodromal period and cognitive and motor decline over years-decades. By contrast, MND is clinically and genetically heterogeneous. Pathogenic variants in at least 50 genes have been implicated in monogenic MND [8]. In a significant proportion of people with MND, potentially causal variants are identified in more than one gene (known as oligogenic inheritance) [9]. Within a family, people with causal variants in the same gene(s) can present with distinct phenotypes, most commonly frontotemporal dementia in some family members, with MND in others and with strikingly different rates of progression. Reduced penetrance also occurs more commonly in MND when compared with HD. The disease course in MND is typically relatively rapid compared to HD, reducing the time for families to adjust to the diagnosis and discuss inheritance and genetic testing. Family communication about inherited disorders is complex and individuals and families may require significant support to ensure information about the availability of predictive testing reaches at-risk family members [6].

While MND and HD are clinically distinct, the family and psychosocial issues around predictive testing show considerable overlap [10]. In the absence of specific guidelines for MND predictive genetic testing, utilisation of an HD testing pathway seems reasonable. Many of the historical concerns about the potential negative consequences of HD predictive testing also apply to MND predictive testing (e.g. familial distress, discrimination and psychological harm). However, given the distinctly different phenotypes and the variability in presentation we believe that a specific guideline for MND predictive testing is warranted. Such a guideline would assist clinicians in planning appropriate evidence-based services and protect individuals undergoing predictive testing from unintentional harm.

We believe that the above factors mandate special care when counselling people who are seeking predictive testing for a known pathogenic variant in an MND gene. Given the highly specialised nature of genetic counselling and testing for MND, non-specialist clinicians may not be aware of all of the relevant issues. A guideline containing recommendations for a predictive testing protocol for MND could enable clinicians to deliver a consistent standard of genetic counselling akin to current HD protocols. This guideline could contain sufficient detail to enable non-genetics specialists to offer predictive testing in settings where genetics specialists are not available. While the general approach to predictive testing in MND will have considerable overlap with HD, there will be multiple gene specific issues around which to base recommendations.

In the first instance, a predictive testing guideline for MND could be produced by an expert working group familiar with existing research and clinical care. This working group could include patient and caregiver representatives (including those who have undergone genetic testing), Clinical Geneticists, Genetic counsellors, Neurologists, healthcare researchers with expertise in developing interventions supporting informed, value-based healthcare decisions (psychology, sociology). However, we recognise the need for future research to produce evidence to underpin and evaluate predictive testing guidance and to implement guidelines into clinical care. Increased understanding of the genetic modifiers of phenotype and penetrance will be crucial to inform gene specific counselling. Expanding our understanding of the ways in which people make decisions to have a predictive test, their information and support needs and preferences for service delivery will guide service design. Long term studies of psychological outcomes in those who undergo testing should be considered to evaluate the impact of the testing process on peoples’ lives. Changes to clinical practice, such as introducing new genetic testing pathways, can take considerable time to be adopted. The time to develop guidelines is now to ensure that systems are in place which may be more easily, and consistently, updated to manage potential increased demands for testing associated with novel therapeutics introduced to treat MND at the presymptomatic stage.
